# Discounting health and money: New evidence using a more robust method

**DOI:** 10.1007/s11166-018-9279-1

**Published:** 2018-05-09

**Authors:** Arthur E. Attema, Han Bleichrodt, Olivier L’Haridon, Patrick Peretti-Watel, Valérie Seror

**Affiliations:** 1 Erasmus School of Health Policy & Management, Erasmus University Rotterdam, Rotterdam, The Netherlands; 2 Erasmus School of Economics, Erasmus University Rotterdam, Rotterdam, The Netherlands; 3 Research School of Economics, Australian National University, Canberra, Australia; 4 CNRS, CREM-UMR 6211, University of Rennes, F35000 Rennes, France; 5 IRD, AP-HM, SSA, VITROME, IHU-Méditerranée Infection, Aix-Marseille University, 13005 Marseille, France

**Keywords:** Time preference, Health versus money, Field study, Direct method, Constant discounting, Hyperbolic discounting, C91, D12, D91, I10

## Abstract

This study compares discounting for money and health in a field study. We applied the direct method, which measures discounting independent of utility, in a representative French sample, interviewed at home by professional interviewers. We found more discounting for money than for health. The median discount rates (6.5% for money and 2.2% for health) were close to market interest rates, suggesting that at the aggregate level the direct method solves the puzzle of unrealistically high discount rates typically observed in applied economics. Constant discounting fitted the data better than the hyperbolic discounting models that we considered. The substantial individual heterogeneity in discounting was correlated with age and occupation.

**Electronic supplementary material:**

The online version of this article (10.1007/s11166-018-9279-1) contains supplementary material, which is available to authorized users.

## Introduction

Many decisions undertaken now will not have consequences until some point in the future. Examples include saving for retirement, getting screened for cancer, and reducing CO2 emissions. When the consequences of an action are further away in the future, people lower their valuation of those consequences. The rate at which future consequences are devalued is called the discount rate.

An important question for both research and policy is whether discount rates depend on the outcome domain. Most research on discounting has used monetary outcomes and it would be very useful if the results from this rich literature could also be used to inform preferences in other domains. Government policy typically uses the same discount rate across domains. For example, the National Institute of Clinical Excellence (NICE), which guides health policy in the UK, discounts the costs and benefits of medical interventions at the same rate (3.5%). The theoretical rationale for using the same discount rate for health and money is questionable (Claxton et al. 2011). Health is less tradeable across time than money and several reasons for discounting money (e.g. inflation, economic growth) are less relevant for health. Similar questions arise regarding the discounting of other non-monetary consequences like environmental goods.

Moore and Viscusi (1990, p.52) argued that the question of whether the discount rate is domain-specific should be resolved empirically. Unfortunately the empirical literature gives no clear answer either. Most existing studies compared the discounting of health and money. While Moore and Viscusi (1990) and Cropper et al. (1994) found the same discount rates for health and money, Cairns (1992) found more discounting for money, and Cairns (1994) and Hardisty and Weber (2009) found more discounting for health gains and less for health losses. Moreover, the correlation between discounting for health and discounting for money was typically low (Chapman and Elstein 1995; Chapman 1996).

A problem in measuring discounting is how to separate discounting and utility. These two components interact, which complicates their measurement. The aforementioned studies avoided this problem by imposing simplifying assumptions on utility. These assumptions may have affected the measured discount rates. Most studies assumed linear utility. However, if utility is concave, which is commonly observed and assumed in economic studies, then the assumption of linear utility can lead to overestimations of discount rates. An additional problem occurs when utility curvature differs across domains. Wakker and Deneffe (1996) found more concave utility for life duration than for money. This difference in utility curvature results in a higher observed discount rate for health than for money even when the actual discount rates are the same.

The aim of our study is to shed new light on the question of whether discounting is domain-specific. We concentrate on the discounting of health and money. We measure discounting by the direct method, recently introduced by Attema et al. (2016). The direct method can measure discounting without the need to measure utility. Consequently, utility can be entirely general and our measurements are not biased by assumptions or measurements of utility. Even if utility differs between health and money, this will not affect our measurements.

We applied the direct method in a large representative sample of the Paris population aged between 30 and 50 years. Our study was commissioned by the French Institute for Health Promotion and Health Education (INPES), and the French Institute for Medical Research (INSERM). Subjects were interviewed at their homes by professional interviewers to obtain high-quality data. Because we used a representative sample, we could investigate whether discounting was related to socio-demographic variables. The literature suggests that such a relation exists, but the findings are equivocal and may also depend on the domain under study. For instance, Table 1 gives an overview of the mixed results on the relation between gender and discounting. For money, several studies found that men were more impatient than women, whereas others found that men were less impatient than women, and yet others found no relation between gender and time discounting. For health the evidence, while less extensive, is equally mixed.

**Table 1:** Empirical evidence on the relation between gender and discounting

Domain	Women more patient	No gender effect	Men more patient
Money	Meier and Sprenger (2010) Ubfal (2016)	Harrison et al. (2002) Anderson and Stafford (2009)	Reynolds et al. (2006) Scharff and Viscusi (2011) Enzler et al. (2014)
Health		Cropper et al. (1992) Bosworth et al. (2015)	Attema and Brouwer (2012)

Finally, our data allow drawing some inferences about the descriptive validity of discount models. Observed deviations from constant discounting, the traditional and still most widely-used discount model, led to the development of a variety of new discount models. Most of these models imply that discounting is not constant but hyperbolic. The available evidence as to which of these hyperbolic models best fits people’s preferences is, again, mixed for both health and money.

Our main findings are as follows. First, we observed that subjects discounted future money more than future health. The medians of the individual discount rates were 2.2% for health and 6.5% for money. These rates are modest compared to the rates that are commonly observed in the empirical literature and they are close to market interest rates. This may be because our measurements were not distorted by utility curvature. It may also be because the direct method is more suitable to express modest discount rates than the methods that are commonly used to measure discounting. This is in spite of the fact that we used a choice-based procedure to measure discounting, which usually gives higher discount rates than directly asking subjects for their indifference values (Ahlbrecht and Weber 1997; Frederick 2003; Read and Roelofsma 2003; Freeman et al. 2016).

There was substantial heterogeneity in discounting, which was correlated with age and, to a lesser extent, occupation. The relation between age and discounting was U-shaped with people around 40 having the lowest discount rates. Finally, and perhaps surprisingly, constant discounting gave a better fit to our data for both money and health than any of the hyperbolic alternatives that we compared.

## Theory

We assume a *preference relation* ≽ over *outcome profiles* (*x*_1_, …, *x*_*T*_) giving *outcome x*_*t*_ at time point *t*.^1^ *T* is a constant denoting the final period. Strict preference and indifference are denoted by ≻ and ∼, respectively. Preferences over outcomes can be derived from preferences over constant outcome profiles (*x*_1_, …, *x*_*T*_) with *x*_*s*_ = *x*_*t*_ for all *s*, *t* ∈ {1, …, *T*}. In our experiment, described in Section 4, outcomes were either health states or monetary amounts. We assume that the decision maker evaluates outcome profiles (*x*_1_, …, *x*_*T*_) by *discounted utility*:

1 $$ {\sum}_{t=1}^T{d}_tU\left({x}_t\right), $$

where *U* is the *utility function* and *d*_*t*_ is the (positive) *discount factor* of time point *t*.

For *E* ⊂ {1, …, *T*}, *α*_*E*_*β* denotes the profile that gives *α* in all time points that belong to *E* and *β* otherwise. Let $C(E)={\sum}_{t\in E}{d}_t$. The discounted utility of profile *α*_*E*_*β* can then be written as *C*(*E*)*U*(*α*) + *C*(*E*^*c*^)*U*(*β*). The term *C*(*E*) reflects the total time weight of period *E*. We write *C*(*k*) = *C*(1, …, *k*). *C* is the *cumulative (discount) weighting function.* We normalize *C* such that *C*(0) = 0 and *C*(*T*) = 1, which is allowed by the uniqueness properties of discounted utility. It is clear from the definition of *C* that once we know *C* we can obtain the discount factors *d*_*t*_ and vice versa.

## Direct method

### Measurements

In our measurements we only used two-outcome profiles *α*_*E*_*β* with *α* ≻ *β*. The first step in the direct method is to elicit the time point *t*_.5_ such that ${\alpha}_{\left[0,{t}_{.5}\right]}\beta \sim {\alpha}_{\left[{t}_{.5},T\right]}\beta$. It follows from Eq. (1) and the definition of the cumulative weighting function *C* that:

2 $$ C\left({t}_{.5}\right)U\left(\alpha \right)+C\left(\left[{t}_{.5},T\right]\right)U\left(\beta \right)=C\left({t}_{.5}\right)U\left(\beta \right)+C\left(\left[{t}_{.5},T\right]\right)U\left(\alpha \right). $$

Equation (2) and *C*(*t*_.5_) + *C*([*t*_.5_, *T*]) = *C*(*T*) = 1 give:

3 $$ C\left({t}_{.5}\right)=C\left(\left[{t}_{.5},T\right]\right)=0.5. $$

Equation (3) shows that the direct method can measure cumulative weights and, consequently, discount rates, without the need to know anything about utility. Utility can be entirely general.

Using *t*_.5_, the time point that has a cumulative weight of 0.5, we can proceed to measure *C* up to any desired degree of precision. In our experiment, we measured five points of *C*. After the elicitation of *t*_.5_, we measured *t*_.25_ from the indifference ${\alpha}_{\left[0,{t}_{.25}\right]}\beta \sim {\alpha}_{\left[{t}_{.25},\kern0.75em {t}_{.5}\right]}\beta$. By a similar argument as above, this indifference gives:

4 $$ C\left({t}_{.25}\right)=C\left(\left[{t}_{.25},{t}_{.5}\right]\right)=0.25. $$

We measured *t*_.125_ by eliciting the indifference ${\alpha}_{\left[0,{t}_{.125}\right]}\beta \sim {\alpha}_{\left[{t}_{.125},{t}_{.25}\right]}\beta$. It follows that *C*(*t*_.125_) = .125. To measure *t*_.75_ we elicited the indifference ${\alpha}_{\left[{t}_{.5},\kern0.75em {t}_{.75}\right]}\beta \sim {\alpha}_{\left[{t}_{.75},T\right]}\beta$, which implies:

5 $$ C\left(\left[{t}_{.5},{t}_{.75}\right]\right)=C\left(\left[{t}_{.75},T\right]\right). $$

Because we know from Eq. (3) that *C*([*t*_.5_, *T*]) = 0.5, it follows that *C*([*t*_.5_, *t*_.75_]) = 0.25 and, by Eq. (1), *C*(*t*_.75_) = *C*(*t*_.5_) + *C*([*t*_.5_, *t*_.75_]) = 0.5 + 0.25 = 0.75. Finally, we measured *t*_.875_ by eliciting the indifference ${\alpha}_{\left[{t}_{.75},{t}_{.875}\right]}\beta \sim {\alpha}_{\left[{t}_{.875},T\right]}\beta$ from which we obtain *C*(*t*_.875_) = 0.875. The above exposition shows that the general principle underlying the direct method is to elicit subjective midpoints of time intervals and to use these to measure the cumulative weighting function *C*.

### Discounting

The discount factors can directly be computed from the cumulative weighting function. For a given *t*_*j*_, Eq. (1) implies $C\left({t}_j\right)={\sum}_{t=1}^{t_j}{d}_t$. The direct method can measure the discount factors nonparametrically, i.e. without making any assumptions about the shape of the discount function. Of course, it can also be used for parametric estimations. The most widely-used discount model is constant discounting for which *d*_*t*_= (1 + *δ*)^−*t*^, with *δ* > 0 the discount rate. Constant discounting can be estimated through the following exponential discount function (see the Online Appendix for details):

6 $$ C\left({t}_j\right)\cong \frac{1-\exp \left(-\delta {t}_j\right)}{1-\exp \left(-\delta T\right)}. $$

Empirical evidence suggests that people systematically deviate from constant discounting and that discount rates usually decrease over time. Several models have been proposed to capture such decreasing impatience. The most popular of these models is quasi-hyperbolic discounting (Phelps and Pollak 1968; Laibson 1997). Other examples include Mazur’s proportional discounting model (Mazur 1987), Harvey’s power discounting model (Harvey 1995), and Loewenstein and Prelec’s generalized hyperbolic discounting model (Loewenstein and Prelec 1992). Ebert and Prelec (2007; see also Bleichrodt et al. 2009) proposed the unit invariance discount function, which can account for both decreasing and increasing discount rates.

The descriptive validity of these discount models is unclear. Table 2 gives an overview of several studies that compared the fit of discount models. Most studies compared only a subset of the abovementioned discount models, but constant discounting was always amongst the models that were compared and quasi-hyperbolic discounting was in most cases.

**Table 2:** Empirical evidence on the fit of discounting models

Study	Domain	Best-fitting model
Angeletos et al. (2001), Paserman (2008), Tanaka et al. (2010)	Money	Quasi-hyperbolic
Abdellaoui et al. (2010)	Money	Constant discounting Power discounting
Abdellaoui et al. (2013)	Money	Unit invariance
Franck et al. (2015)	Money	Generalized hyperbolic discounting
Kirby (1997)	Money	Proportional discounting
Keller and Strazzera (2002)	Money	Power discounting
Andreoni and Sprenger (2012), Attema et al. (2016)	Money	Constant discounting
van der Pol and Cairns (2002)	Health	Generalized hyperbolic discounting Power discounting
Bleichrodt and Johannesson (2001) van der Pol and Cairns (2011)	Health	Generalized hyperbolic discounting
Bleichrodt et al. (2016)	Health	Generalized hyperbolic discounting Proportional discounting

Table 2 shows that the best-fitting discount model varied across studies. Most studies found deviations from constant discounting, but they give equivocal results about which alternative to use. Note that some studies actually found that constant discounting fitted at least as well as alternative discount models.

## Experiment

### Subjects

We recruited 505 subjects representative of the population in the Paris region. Because our experiment asked subjects to make tradeoffs involving health and money in 20 years, we only recruited subjects between the age of 30 and 50 years. Younger people may have no stable income, making it hard to imagine their income over the next 20 years and to make the required tradeoffs. Older people may find it difficult to project themselves in 20 years’ time. Participation in the experiment was voluntary and no incentives or rewards were offered. We discuss the issue of incentives in Section 5.

### Procedure

A professional sampling company (BVA company) programmed and conducted the experiment. Subjects were contacted by phone and, if they agreed to participate, interviewed at home by professional interviewers. We used face-to-face interviews to get high-quality data. The protocol was tested in two pilot sessions. After the pilot sessions there was a feedback session where the interviewers gave their comments and asked questions about the experiment and we adjusted the experiment based on these comments and questions. A copy of the actual experiment can be found in the Online Appendix (Appendix A).

The experiment was computer-run. Responses were entered by the interviewers to reduce errors. Subjects were first informed about the goal of the study (to assess their attitudes towards quality of life, health, and time), the organizers (INPES and INSERM), the poll company, and the legal conditions of the interview (mostly about the anonymity of their answers). They then received instructions. When the subjects had completed the experiment, they were asked some socio-demographic questions, questions about their households’ financial situation, and whether they (had) suffered from back pain, which is the health state used in the experiment. The experiment was part of a larger questionnaire.

### Design

We used both health and monetary profiles. For health, subjects were told to imagine that they suffered from a mild but continuous back pain. Back pain was described by the EQ-5D system, which is widely used in medical research. It describes health states by their scores on five dimensions with three levels each. The description of back pain is in the experimental instructions, which are in the Online Appendix. We told subjects that back pain could be treated by taking a weekly dose of pills, which would result in full health. In the notation of Section 3, back pain corresponded to outcome *β* and full health to outcome *α*. Asking subjects to trade off periods in which their health changes is common in health economics. This requires some abstract thinking by subjects, which might lead to noise. This is a limitation that the direct method shares with traditional methods to measure discounting in health. On the other hand, the direct method has some advantages over these traditional methods as explained below and in more detail in Attema et al. (2012).

For money, subjects had to imagine that their purchasing power would improve by 20%. Consequently, monetary amount *β* corresponded to the subject’s current purchasing power and monetary amount *α* to the 20% increase in purchasing power. We framed the questions in terms of purchasing power to control for subjective differences in perceived and expected future inflation. Similarly, we used the subject’s current purchasing power as outcome *β* to control for differences in future wealth expectations. By using purchasing power we could frame both health and money profiles as continuous flows and make the choices in the two domains appear similar.

The health and money profiles involved 20 years in total. After these 20 years, all health profiles resulted in the same health outcome and all money profiles in the same monetary outcome. In the direct method this common outcome can be left unspecified. Traditional methods to measure discounting have to specify what happens after the period under consideration. For example, most methods to measure discounting for health tell subjects that the profiles end in death. They thus have to specify the exact time of death, which is clearly unrealistic, and has been a major limitation of health investigations.

The order of the health and money questions was randomized. To avoid confusion, we did not intersperse the health and money questions and we only moved to the health [money] questions when all the money [health] questions had been answered.

The nature of the direct method imposed that we first had to elicit *t*_.5_. Using this time point, we then elicited *t*_.25_ and *t*_.75_, which in turn were used to elicit *t*_.125_ and *t*_.875_. The order in which *t*_.25_ and *t*_.75_ were elicited and the order in which *t*_.125_ and *t*_.875_ were elicited was randomized between subjects.

**Fig. 1:**
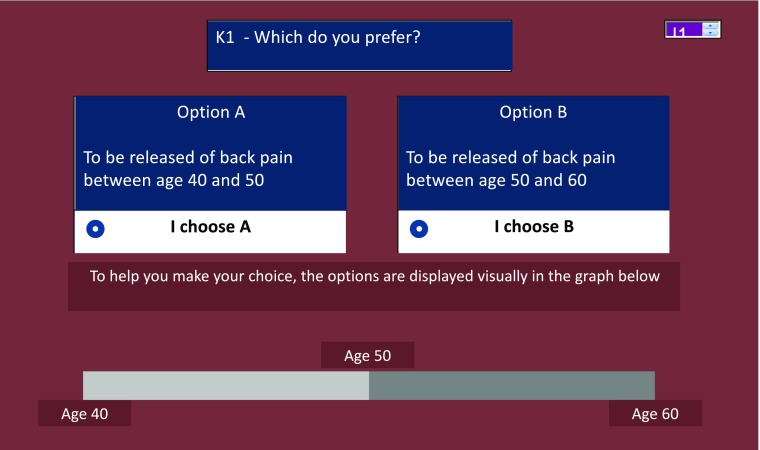
Example of a choice question

Figure 1 shows the presentation of the health questions.^2^ An example of the money questions is in the Online Appendix. Subjects chose between two options. The left-hand option (Option A) always started with the improvement in health, the right-hand option (Option B) always ended with the improvement in health. Subjects clicked on their preferred option and the stimuli were then adjusted to make the chosen option less attractive and the non-chosen option more attractive. We used a choice-based elicitation procedure because it leads to more reliable measurements (Bostic et al. 1990) and to discount rates that are more closely associated with real-world behavior (Hardisty et al. 2013) than directly asking subjects for their indifference values.

The change in the stimulus values was halved after each switch in preference. The pilot sessions showed that subjects found it difficult to choose when life duration was expressed in months and we therefore only used years as the unit of time. The elicitation ended when the change in the stimulus values was less than 1 year. Table 3 gives an example for a 40-year-old subject. In the table, *α*_[*s*, *t*]_*β* means to get *α* between age *s* and age *t* and *β* at all other ages. The option that the subject chose is printed in bold. We set the indifference value equal to the midpoint between the smallest value for which the left-hand profile was preferred and the largest value for which the right-hand profile was preferred. In Table 3 this midpoint is 43.5 years. To control for response errors, we repeated the first choice at the end of the elicitation. If the subject made the same choice in the repeated choice, he moved on to the next question. If not, the choice-based procedure was started anew.^3^ In the analysis we used the data from the elicitation for which the subject made the same choice in the repeated question.

**Table 3:** Illustration of a choice-based elicitation for a 40-year-old subject

***Step***	***Choices***
*1*	***α***_[**40**, **50**]_***β*** vs. *α*_[50, 60]_*β*
*2*	***α***_[**40**, **45**]_***β*** vs. *α*_[45, 60]_*β*
*3*	*α*_[40, 43]_*β* vs. ***α***_[**43**, **60**]_***β***
*4*	***α***_[**40**, **44**]_***β*** vs. *α*_[44, 60]_*β*
*Indifference Value*	*43.5*

### Analysis

Besides classical statistical tests, we also computed Bayes factors to assess the support for the various hypotheses. We follow the convention of concluding that there is some evidence for a hypothesis if the Bayes factor exceeds 3, that there is strong evidence if the Bayes factor exceeds 10, and that there is very strong evidence if the Bayes factor exceeds 30 (Jeffreys 1961).

Some subjects did not complete all questions because the remaining intervals were too narrow to allow eliciting new values. If the interval [0,*t*_.5_] was too narrow, which happened when subjects always chose Option A in Figure 1 and were extremely impatient, then we set *t*_.125_ = *t*_.25_ = *t*_.5_ = 0. These subjects had an annual discount rate exceeding 69%. If the interval [ *t*_.5_, 1] was too narrow, which happened when subjects always chose Option B in Figure 1 and were extremely patient, we set *t*_.875_ = *t*_.75_ = *t*_.5_ = 20. For these subjects the annual discount rate went to minus infinity. If the interval [0, *t*_.25_] was too narrow we set *t*_.125_ = *t*_.25_ = 0. Finally, if the interval [*t*_.75_,1] was too narrow we set *t*_.875_ = *t*_.75_ = 20. We also analyzed the results by excluding the subjects with such extreme preferences. As there were more extremely impatient subjects than extremely patient subjects this decreased overall discounting, but it did not affect our main conclusions about the relation between discounting for health and money, the fit of the discount models, and the effect of the socio-demographic variables on discounting.

We did not allow non-integer stimuli as this would make the questions very cognitively demanding. For example, in Table 1 we elicited *t*_.5_ = 43.5, but then 44 was used in the elicitation of *t*_.25_ and *t*_.75_. We used two strategies to account for this rounding. The first strategy assumed that all values were determined by three indifferences only. Then no rounding occurred but the indifference values were determined somewhat less accurately. The second strategy assumed that *t*_.25_, *t*_.5_, and *t*_.75_ were determined by four iterations and rounded to the nearest integer greater than or equal to that value. For *t*_.125_ and *t*_.875_ the rounding problem did not occur and we could use the value that was determined after four iterations. The two analyses led to the same conclusions about the relationship between the discounting of health and money. We will only report the latter, because it uses more data points. Detailed information about the rounding strategies is in the Online Appendix.

The area under the cumulative weighting function indicates the degree of discounting. The larger this area, the more a subject discounts the future. We normalized the cumulative weighting functions *C* for health and money by dividing the *t*_*j*_ by 20. This ensured that the area under the curves was between 0 and 1. If *C* is linear, the area is ½. Values exceeding ½ correspond to concavity of *C* and positive discounting, and values less than ½ correspond to convexity of *C* and negative discounting. We used linear interpolation to compute the area.

We explored the fit of various discount models. We had only five data points per subject and, hence, two-parameter models^4^ often did not converge. We therefore estimated the following one-parameter models^5^:

- Constant discounting: *d*_*t*_= (1 + *δ*)^−*t*^
- Proportional discounting: *d*_*t*_= (1 + *κt*)^−1^
- Power discounting: *d*_*t*_= (1 + *t*)^−*α*^
- Dual exponential discounting: *d*_*t*_ = 0.5*e*^−*rt*^ − 0.5*e*^*rt*^ + 1
- Periodic discounting: *d*_*t*_ = 0.5 + 0.5 cos(*ρt*) − 0.5 sin(*ρt*)

Dual discounting and periodic discounting are the special one-parameter cases of two discount models proposed by Prelec and Rohde (2016). They are flexible functional forms that can account amongst other things for decreasing and increasing impatience, a preference for increasing sequences, and preferences that focus primarily at the beginning and the end of sequences of outcomes. In contrast with the other four discount functions, in periodic discounting the cumulative weighting function was normalized as *t* = *t*_*j*_ ∗ *π*/20. We estimated the discount models using a continuous approximation (see the Online Appendix for details) and a nonlinear least squares procedure.

Finally, we explored the relations between discounting and socio-demographic characteristics. We used Tobit regressions because the areas under the normalized weighting functions were censored between 0 and 1. Because of the 20-year age difference between the youngest and the oldest of our subjects, we corrected for the general increase in educational attainment over time. We categorized educational level into four classes corresponding to the International Standard Classification of Education. Subjects’ relative educational positions were then defined as the mean proportion, by 5-year age group, of subjects with an educational level higher than theirs (Mackenbach et al. 1997).^6^ We also accounted for subjects’ current experience of back pain and the order in which the tasks were presented. We ran three Tobit regressions. The first two regressions, Model I and Model II, regressed discounting for health and money separately on the set of explanatory variables. The third regression, Model III, pooled discounting for health and money together to have another test of whether discounting was domain-specific.

## Results

Table 7 in the Appendix shows the summary statistics of our sample. In particular, 36.6% of the subjects were currently suffering from back pain (the health state used in the experiment), and 17.0% reported having a monthly income below €1500.

### Cumulative weighting functions

**Fig. 2:**
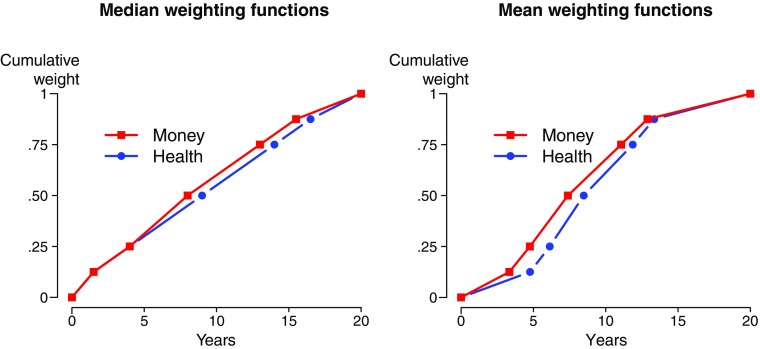
Median and mean cumulative weighting functions for health and money

Figure 2 shows the median and mean cumulative weighting functions for health and money. A table with descriptive statistics is in the Appendix (Table 8). Figure 2 shows that the cumulative weighting function for money was above the cumulative weighting function for health. This indicates more discounting for money than for health. Statistical tests confirmed that the elicited values of *t*_*j*_ differed significantly between health and money (ANOVA with repeated measures, *p* < 0.01). A Bayesian ANOVA led to very strong support for the hypothesis that the time weights differed between health and money (Bayes factor (BF) = 155.49). In three of the five tests we observed some support for the hypothesis that the time weights for health were higher than those for money (*BF* > 4.8). For weight t_.75_, the evidence was inconclusive (*BF* = .63). For weight t_.875_, we found some support for the hypothesis that the time weights for health and money were the same (*BF* = .15). Figure D.1 in the Online Appendix shows the cumulative distribution functions of the individual time weights. The distributions for money were to the left of those for health, signaling more discounting for money.

The direct method gives a convenient nonparametric measure of the degree of discounting by computing the area under the cumulative weighting function *C*. The median areas were 0.54 for health and 0.57 for money, which indicate positive discounting. Both areas were significantly different from 0.50, the case of no discounting (Wilcoxon test, both *p* < 0.01).^7^ For health, 274 [190, 31] subjects showed positive [negative, no] discounting. For money this was true for 324 [117, 54] subjects. The proportion of subjects with positive discounting was significantly higher than the proportion of subjects with negative discounting. (Binomial test, both *p* < 0.01).^8^

The area measure confirmed that subjects discounted money more than they discounted health. The area under the curve was significantly larger for money than for health (Wilcoxon test, *p* < 0.01).^9^ For 234 [172] subjects, the area under the curve for money was larger [smaller] than the area under the curve for health. The proportion of subjects for whom the area under the curve was larger for money was significantly higher than the proportion of subjects for whom it was larger for health (Binomial test, *p* < 0.01, *BF* > 10^6^).

**Fig. 3:**
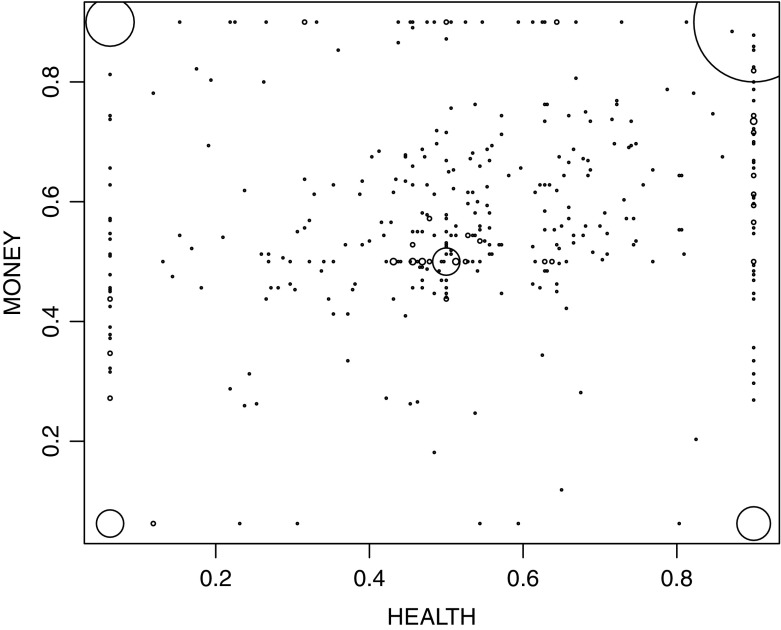
Relation between the area measures for health and money

Figure 3 shows the relation between the area measures for health and money. The size of the dots reflects the number of data points. The correlation between discounting for health and discounting for money was fair (Kendall’s *τ* = 0.22). It is similar to most of the correlation coefficients observed by Hardisty and Weber (2009) for money, health, and environmental goods. Discounting was largely domain-specific although the correlations were significant and there appears to be a common component in time preferences. The relatively low correlation was not caused by extreme answers. When we removed the extreme answers Kendall’s *τ* only increased slightly, to 0.25.

### Discount models

We also fitted the cumulative weighting functions by five parametric forms to explore which discount function best described our subjects’ preferences. Table 4 shows the medians of the individual estimates of the parameters in each of the models. The parameter *δ* in constant discounting is equal to the discount rate. We found median estimated discount rates of 2.2% for health and of 6.5% for money.^10^ These rates are much lower than what has usually been observed in the literature. For health they are close to the rates observed by Attema et al. (2012) who also used the direct method.

**Table 4:** Estimated discount functions

	Constant discounting	Proportional discounting	Power discounting	Dual exponential discounting	Periodic discounting
Health	*δ* = 0.022 [−0.063, 0.392]	*κ* = 2849.7 [0.794, 2849.7]	*α* = 0.467 [−0.16,2.285]	*r* = 0.097 [0.00,0.172]	*ρ* = 0.183 [−0.184,1.084]
Money	*δ* = 0.065 [0.00, 0.298]	*κ* = 2.404 [0.226, 2849.7]	*α* = 0.493 [0.00,1.381]	*r* = 0.108 [0.083,0.137]	*ρ* = 0.304 [0.00,0.722]

The table shows the medians of the individual estimates with the interquartile range (IQR) in square brackets

**Fig. 4:**
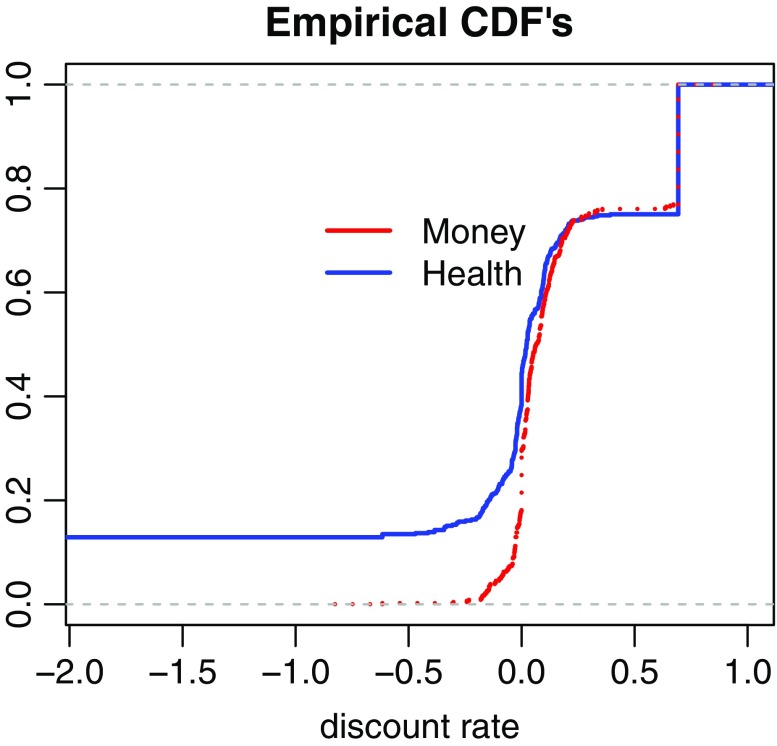
Cumulative distribution functions of the discount rates for health and money

Figure 4 shows the cumulative distribution functions of the discount rates for health and money. The distribution for health lies mostly above the distribution for money, which indicates more discounting for money than for health. The figure shows that most subjects had discount rates close to zero for both health and money. This suggests that not only at the aggregate level but also for most individual subjects the direct method elicits more reasonable discount rates than other methods that have been used in the literature.

A substantial proportion of our subjects had negative discount rates (around 44% for health and around 30% for money). Negative discounting has been observed before for sequences of outcomes: people tend to prefer increasing sequences over decreasing ones (Loewenstein and Prelec 1993; Manzini et al. 2010). For health, negative discount rates have quite frequently been observed (MacKeigan et al. 1993; Redelmeier and Heller 1993; van der Pol and Cairns 2000; van der Pol et al. 2015).

Table 5 shows that constant discounting gave the best fit for 49.7% of the subjects for health and for 49.4% of the subjects for money. Of the hyperbolic models, power discounting gave the best fit. In spite of its flexibility, periodic discounting did not perform particularly well, especially for health. For 27.9% of the subjects, constant discounting fitted best for both health and money. For the other models this proportion was less than 5%. For most subjects the best-fitting model varied across the two domains.

**Table 5:** Proportion of subjects for whom each of the discount models fitted best

	Constant	Proportional	Power	Dual exponential	Periodic
Health	49.70%	4.21%	17.44%	18.64%	10.01%
Money	49.4%	6.00%	14.6%	6.0%	24.0%

Goodness of fit was measured in terms of the residual sum of squared errors

### Time discounting and socio-demographic variables

Table 6 shows the results of the Tobit regressions, which explain the area under the normalized cumulative weighting functions for health and money by a set of socio-demographic variables.

**Table 6:** The effect of socio-demographic variables on discounting

	Area under the normalized utility function					
	Health		Money		Pooled	
	Estimates (SE)	*p*-values	Estimates (SE)	*p*-values	Estimates (SE)	*p*-values
Monetary outcomes (ref = health outcomes)					0.051 (0.023)	0.028
Men (ref = no)	−0.029 (0.041)	0.482	−0.042 (0.03)	0.164	−0.036 (0.025)	0.149
Age	−0.086 (0.045)	0.059	−0.092 (0.033)	0.006	−0.089 (0.028)	0.001
Age^2^	0.001 (0.001)	0.048	0.001 (0)	0.005	0.001 (0)	0.001
Couple (ref = no)	−0.036 (0.053)	0.501	−0.003 (0.039)	0.93	−0.017 (0.033)	0.596
Children (ref = no)	−0.017 (0.054)	0.752	−0.005 (0.039)	0.899	−0.012 (0.033)	0.722
Relative educational position	0.145 (0.084)	0.084	−0.039 (0.061)	0.529	0.046 (0.051)	0.371
Currently employed (ref = no)	−0.006 (0.054)	0.905	0.063 (0.04)	0.113	0.032 (0.033)	0.338
Public sector (ref = no)	−0.049 (0.048)	0.303	−0.071 (0.035)	0.039	−0.062 (0.029)	0.032
At least one manual or service employee in the household ^1^ (ref = no)	−0.157 (0.048)	0.001	0.038 (0.035)	0.278	−0.053 (0.029)	0.068
Low-income household	0.01 (0.058)	0.865	0.043 (0.043)	0.313	0.029 (0.036)	0.424
Suffering from back pain (ref = no)	0.057 (0.04)	0.16	−0.023 (0.03)	0.44	0.015 (0.025)	0.54
Back pain related scenario first	0.02 (0.039)	0.606	0.032 (0.028)	0.261	0.026 (0.024)	0.279
Constant	2.184 (0.887)	0.014	2.363 (0.654)	<0.001	2.255 (0.546)	<0.001
McFadden adjusted R^2^	0.031		0.039		0.024	

Tobit regression, with left-censored values at 0.0625 and right-censored values at 0.9

The goodness of fit was low as shown by the McFadden adjusted R^2^. For health, the only variables that were related to discounting were age, occupation, and (marginally) education. Subjects who performed physically demanding occupations had lower discounting (*p* = 0.001). This is somewhat counterintuitive as physically demanding occupations may lead to more rapid decreases in and more uncertainty about health and, hence, people who hold these occupations may care less about their future health. However, it could also be that these people are relatively healthy so they expect no decreases in their health in the future. Subjects with a lower educational position had marginally higher discounting (*p* = 0.08). The relation between age and discounting was U-shaped with people around the age of 40 having the lowest rate of health discounting. We observed no significant effect of suffering from back pain on discounting.

For money, only occupation and age were significant. Working in the public sector (characterized by a high level of employment security) was associated with less discounting (*p* = 0.03). This is plausible as public sector jobs in France usually come with a high level of employment security and discounting can at least partly be explained by uncertainty about the future (Baucells and Heukamp 2011; Epper et al. 2011). As for health, we observed a U-shaped relation between discounting and age with people around the age of 40 having the lowest rate of discount (*p* < 0.01).

The pooled regression showed different discounting for money than for health (*p* = 0.03), confirming that discounting was domain-specific. The pooled regression also confirmed the effects of age and occupation on discounting. Subjects who performed physically demanding occupations had marginally lower discounting (*p* = 0.07) and working in the public sector was associated with less discounting (*p* = 0.04).

## Discussion

Discounting was domain-specific and our subjects discounted money more than health. There was only a fair correlation between discounting for money and discounting for health. In many countries governments use the same discount rates for money and health.^11^ Our findings suggest that this is not consistent with people’s preferences. If government policy aims to reflect people’s preferences, then it should discount health and money differently. Some countries already discount the costs and benefits of public health programs at different rates and our results support this practice.^12^

Why did our subjects discount money more than health? One answer is that it may be rational to do so. Several reasons why people discount money do not apply (or apply less) to health. For example, the growth rate of GDP tends to be larger than the growth rate of health,^13^ which means that the value of health in terms of consumption increases and which may justify a lower discount rate for health (Van Hout 1998; Gravelle and Smith 2001; Claxton et al. 2011). Indeed, empirical evidence suggests that the value of a statistical life increases with income and, hence, increases over time as income grows (Viscusi and Aldy 2003; Hammitt and Robinson 2011).

The median discount rates and also most of the individual discount rates that we observed were reasonable and close to market interest rates. This is particularly noteworthy as we used a choice-based elicitation method, which generally leads to higher discount rates. The observed lower discount rates are an argument in favor of the direct method. Most of the literature on intertemporal preferences has observed high discount rates, typically well above 20%. It is hard to reconcile such high discounting with the interest rates observed in the financial markets and hence it is hard to defend their status, particularly in prescriptive analyses.

Why does the direct method lead to more reasonable discount rates? One reason is that it is not affected by assumptions about utility. Most studies assume linear utility and this leads to an upward bias in estimated discount rates if utility is concave. A second reason may be that the direct method, by using sequences of outcomes, makes it easier to express lower rates of discounting than the methods that are commonly used to measure discounting. The common approach to measure discounting is to ask subjects to trade off a smaller amount sooner against a larger amount later. For example, Hardisty and Weber (2009) asked their subjects which amount *X* in 1 year they consider equivalent to $250 now. A discount rate of 5% implies *X* = *$*262.50. However, most subjects perceive the difference between $250 and $262.50 as negligible whereas waiting 1 year matters. Such similarity-based reasoning can lead to the high discount rates that are commonly observed. The direct method is not affected by this effect. Read et al. (2005) showed that the framing of discounting questions matters. Frederick et al. (2008) found markedly lower discount rates when delays were described in terms of the subjects’ age than when they were described as time intervals. Our findings are consistent with this effect.

A second finding of our study was that for around half of the subjects constant discounting gave a better fit to their data than any of the hyperbolic models that we considered for both health and money. This may be surprising given that many studies have found violations of constant discounting (Frederick et al. 2002; Attema 2012). It is consistent with Attema et al. (2016) who also used the direct method for money. They hypothesized that constant discounting may have given the best fit in their study because they did not include the present. Most violations of constant discounting have been found for the present because of the immediacy effect. Our data included the present, but constant discounting still gave the best fit. Of course, we only compared constant discounting with other one-parameter discount models (as the two-parameter models did not converge well). Attema et al. (2016) found that constant discounting fitted better than generalized hyperbolic discounting (Loewenstein and Prelec 1992) and unit invariance discounting (Ebert and Prelec 2007; Bleichrodt et al. 2009), two-parameter discount functions that performed well in earlier studies. On the other hand, Bleichrodt et al. (2016) found that generalized hyperbolic discounting and proportional discounting fitted better than constant discounting. They used a method that makes no assumptions about utility either.^14^ The question about the best-fitting discount model is still open although our data suggest that constant discounting need not necessarily be rejected.^15^ The direct method can answer this question but it requires collecting more data points than the five that we collected.

Our third finding is that the socio-demographic variables that we included contributed little to the explanation of discounting. The main explanatory variables were age, for which we observed a U-shaped relation, and occupation. People around the age of 40 had the lowest discount rate. It should be kept in mind that our sample only covered the age range 30–50 years. However, the observed U-shaped relation is predicted by the theoretical model of Sozou and Seymour (2003) and is consistent with previous empirical studies that used larger age spans (Read and Read 2004; Enzler et al. 2014). Regarding occupation, we found that subjects with physically demanding jobs discounted health outcomes less and working in the public sector was associated with lower discounting of monetary outcomes.

Previous studies found that better-educated people were more patient (Warner and Pleeter 2001; Harrison et al. 2002; Meier and Sprenger 2013; Enzler et al. 2014; Courtemanche et al. 2015). Our study did not confirm this finding although we found a marginal effect of education on the discounting of health. This difference in findings could be caused by our use of a method that makes no assumptions about utility. Another possibility is that the effect of education disappeared because of an interaction with occupation. The public sector workers in our study were indeed better educated than our average subject. We found no association between income and discounting, unlike several earlier studies (Tanaka et al. 2010; Meier and Sprenger 2013). Likewise we found no effect of marital status or the presence of children. This is consistent with the findings of Enzler et al. (2014). Suffering from back pain had no effect on time discounting, which is inconsistent with the predictions of the theoretical model of Becker and Mulligan (1997) and which seems to contradict the empirical findings of Chao et al. (2009) who found a U-shaped relation between discounting and health. However, it is consistent with Read and Read (2004) who, like us, found no relation between health and the discounting of money.

The direct method makes no assumptions about utility. However, it does assume separability across time, which is a strong assumption. Violations of separability could be caused by sequencing effects or habit formation (Gilboa 1989; Loewenstein and Prelec 1993; Wathieu 1997). On the other hand, Attema et al. (2016) tested separability by the direct method and found that it held for 80% of their subjects.

Our study was commissioned by INPES and INSERM. They wanted to know whether people discounted health and money the same over longer time spans with an eye on the evaluation of prevention programs. Because we used health as an outcome and studied longer time spans, we could only answer their question by using hypothetical choices. Some economists question the use of hypothetical choices and object that they may lead to less careful responses that do not represent subjects’ true preferences. The evidence on the use of real versus hypothetical questions in measuring time preferences is mixed. Several studies observed no difference between real and hypothetical choices (Johnson and Bickel 2002; Madden et al. 2003; Ubfal 2016). The studies that did observe a difference led to mixed conclusions. Kirby and Marakovic (1995) found more discounting in real tasks, whereas Coller and Williams (1999) found less discounting in real tasks. Summing up the available evidence, Frederick et al. (2002, p.389) concluded that “there is, as yet, no clear evidence that hypothetical rewards are discounted differently than real rewards.”

## Conclusion

We have used Attema et al.’s (2016) direct method to investigate whether discounting for money and health are the same. The direct method measures discounting without requiring assumptions about utility. We applied the direct method in a field study using a representative sample of the Paris region of people between age 30 and 50 who were individually interviewed by professional interviewers. Our subjects discounted money more than health. The elicited median discount rates were reasonable: 2.2% for health and 6.5% for money. This suggests that the direct method is able to solve the empirical puzzle of the incredibly high discount rates that are commonly observed in experiments and field studies. At the individual level, we observed the usual heterogeneity in discounting, but for most subjects the elicited discount rates were also close to the market interest rates. Constant discounting fitted our data better for more subjects than any of the hyperbolic models that we investigated for both money and health. Discounting was related to age. The relation was U-shaped with the lowest discounting for subjects around the age of 40. Occupation also contributed to the explanation of discounting. The other demographic variables, including education and income, did not contribute much.

### Electronic supplementary material

ESM 1 (PDF 352 kb)

## Appendix: Descriptive statistics

Table 7 shows the summary statistics of our sample.

**Table 7:** Summary statistics, individual characteristics (*N* = 505)

	Total	
	N	%
Gender		
- men	244	48.3
- women	261	51.7
Age		
mean (standard deviation)		40.00 (6.26)
Married or living as a couple		
- yes	352	69.7
- no	153	30.3
Children		
- yes	358	70.9
- no	147	29.1
Educational level		
- No educational qualifications	41	8.1
- Less than secondary school	153	30.3
- Secondary school graduation	76	15.0
- Above secondary school graduation	235	46.5
Relative educational position		
mean (standard deviation)		0.50 (0.27)
Occupational status		
- Working	401	79.4
- Unemployed	32	6.3
- Other (housewife, parental or other long time leave, retired)	72	14.3
Private/public sector		
- public sector	113	22.4
- private sector	337	66.7
- Not applicable (housewives, students, ...)	55	10.9
Occupational type		
- Manual and service employees	107	21.2
- Office employees	85	16.8
- Craftsmen, salesmen	20	4.0
- Intermediate occupations^a^	101	20.0
- Management and related ^b^	137	27.1
- Not applicable	55	10.9
Household low occupational type (surveyed individuals and/or partners)^c^		
- yes	149	29.5
- no	356	70.5
Household’s monthly income below 1500€		
- yes	86	17.0
- no	419	83.0
Currently suffering from back pain		
- yes	185	36.6
- no	320	63.4
Extreme discounting		
- Health	201	39.8
- Money	156	30.9
Back pain related scenario first presented to subjects	279	55.2

^a^Intermediate occupations were foremen, first-line supervisors, paramedics, and primary school teachers

^b^Management and related occupations were liberal professions (except paramedical), professors/ scientific professions, managers and other intellectual professions

^c^Low occupational type related to manual and service employees. For housewives, occupational type was defined on the basis of their partner’s occupation

Table 8 shows the mean and medians of the elicited values.

**Table 8:** Summary statistics and tests, elicited indifference values

	*t*_.125_	*t*_.25_	*t*_.5_	*t*_.75_	*t*_.875_
Health					
Mean (standard deviation)	4.77 (6.68)	6.13 (6.63)	8.48 (6.89)	11.87 (6.71)	13.36 (6.61)
Median	1.5	4	9	14	16.5
Interquartile range	[0, 5]	[0, 8]	[0, 13]	[4, 17]	[4.5,18.5]
Money					
Mean (standard deviation)	3.34 (5.23)	4.76 (5.27)	7.39 (5.79)	11.07 (6.01)	12.89 (6.16)
Median	1.5	4	8	13	15.5
Interquartile range	[0.5,2.5]	[1, 5]	[3, 10]	[6, 15]	[7.25,17.5]
Wilcoxon tests					
whole sample	*p* < 0.01	*p* < 0.01	*p* < 0.01	*p* = 0.01	*p* = 0.07
Sub-sample of subjects with non-extreme answers (*N* = 259)	*p* < 0.01	*p* < 0.01	*p* < 0.01	*p* = 0.22	*p* = 0.50

## Footnotes

1 In this paper we consider discrete outcome streams. For an extension to continuous outcome streams see Attema et al. (2016).

2 The question has been translated. The actual question, which was in French, is in the Online Appendix.

3 37% of the subjects started a question anew. These subjects did that on average 1.78 times. One hundred and twelve subjects restarted at least one health question and 111 subjects restarted at least one money question. The Pearson correlation between restarting a health question at least once and restarting a money question at least once was poor (*ρ* = 0.11).

4 Examples are quasi-hyperbolic discounting (Phelps and Pollak 1968; Laibson 1997), generalized hyperbolic discounting (Loewenstein and Prelec 1992), and constant sensitivity or unit invariance (Ebert and Prelec 2007; Bleichrodt et al. 2009).

5 These are the only one-parameter discount models that we are aware of.

6 The four educational classes were (1) no education or primary education, (2) lower secondary education, (3) upper secondary education, and (4) post-secondary and tertiary education. This continuous measure decreases with the level of education.

7 Both *BF* > 237.

8 The Bayes factors indicated very strong support for the hypothesis that there were more subjects with a concave than a convex weighting function (*BF* > 10^6^).

9 The data supports the hypothesis that the area is larger for money than for health (Bayes factor = 6.97).

10 Based on the median data the estimated discount rates were 2.7% for health and 4.4% for money.

11 Examples include Australia (5%), Austria (3%), Canada (5%), Croatia (3%), France (4%), Germany (3%), Ireland (5%), Italy (3%), New Zealand (3.5%), Spain (3%), Sweden (3%), the UK (3.5%), and the US (3%).

12 Countries with different discount rates are Belgium (money 3%, health 1.5%), the Netherlands (money 4%, health 1.5%), Poland (money 5%, health 3.5%), and Russia (money 5%, health 0%).

13 As measured for instance by quality-adjusted life-years (Drummond et al. 2015).

14 They did not measure discounting, but deviations from constant impatience.

15 Read et al. (2005) found that hyperbolic discounting performed better than exponential discounting when delays were described in terms of time intervals, but not when they were described in terms of calendar dates. This may suggest a possible explanation for our finding. However, DeHart and Odum (2015) who used a wider range of delays than Read et al. (2005) could not confirm this conclusion and also found more support for the hyperbolic model when delays were described in terms of calendar dates.
