# Supplementary material for: Discounting health and money: New evidence using a more robust method
Source: J Risk Uncertain. 2018 May 9;56(2):117–40. doi: 10.1007/s11166-018-9279-1 (PMC6445504; doi:10.1007/s11166-018-9279-1)
Supplement: Supplementary file 1 — (PDF 352 kb) [file 11166_2018_9279_MOESM1_ESM.pdf]

Discounting Health and Money: New Evidence Using A More Robust Method

Arthur E. Attema[1], Han Bleichrodt [2], Olivier l'Haridon[3], Patrick Peretti-Watel[4], and Valérie Seror[5]

[1] *Erasmus School of Health Policy & Management, Erasmus University Rotterdam, the Netherlands, attema@eshpm.eur.nl*
[2] *Erasmus School of Economics, Erasmus University Rotterdam, the Netherlands Research School of Economics, Australian National University, Canberra, Australia bleichrodt@ese.eur.nl*
[3] *Univ Rennes, CNRS, CREM-UMR 6211, F-35000 Rennes, France, olivier.lharidon@univ-rennes1.fr*
[4] *Aix-Marseille Univ, IRD, AP-HM, SSA, VITROME, IHU-Mditerrane Infection, F-13005, Marseille, France, patrick.peretti-watel@inserm.fr*
[5] *Aix-Marseille Univ, IRD, AP-HM, SSA, VITROME, IHU-Mditerrane Infection, F-13005, Marseille, France, valerie.seror@inserm.fr*

Online Appendix

A Example of choice questions used in the experiment

Figure A.1 shows an example of the display of the money questions. Figure A.2 shows the original version of the choice question displayed in Figure 1 in the main text.

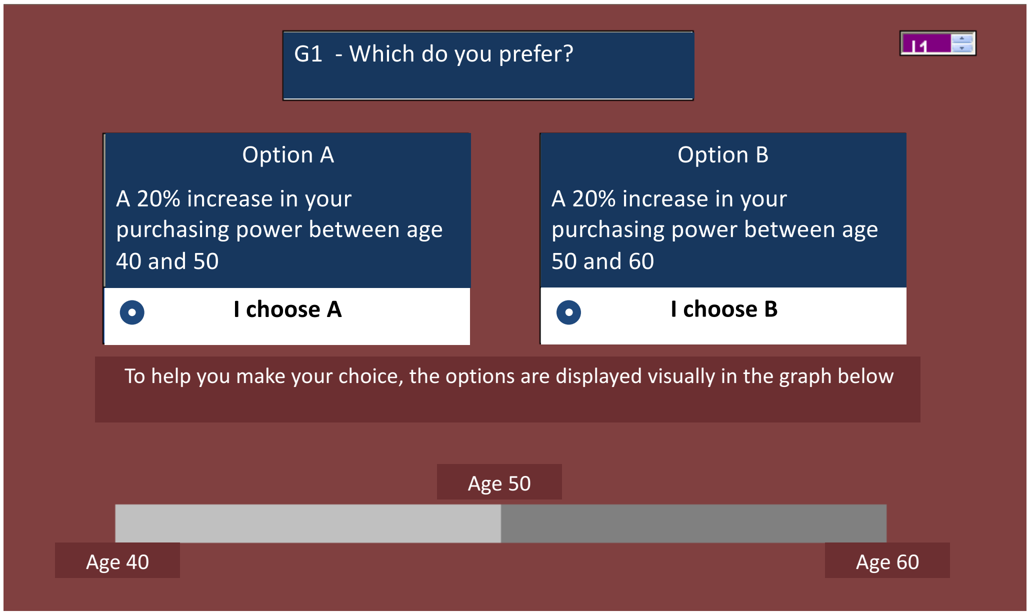

Figure A.1: Example of a choice question for money

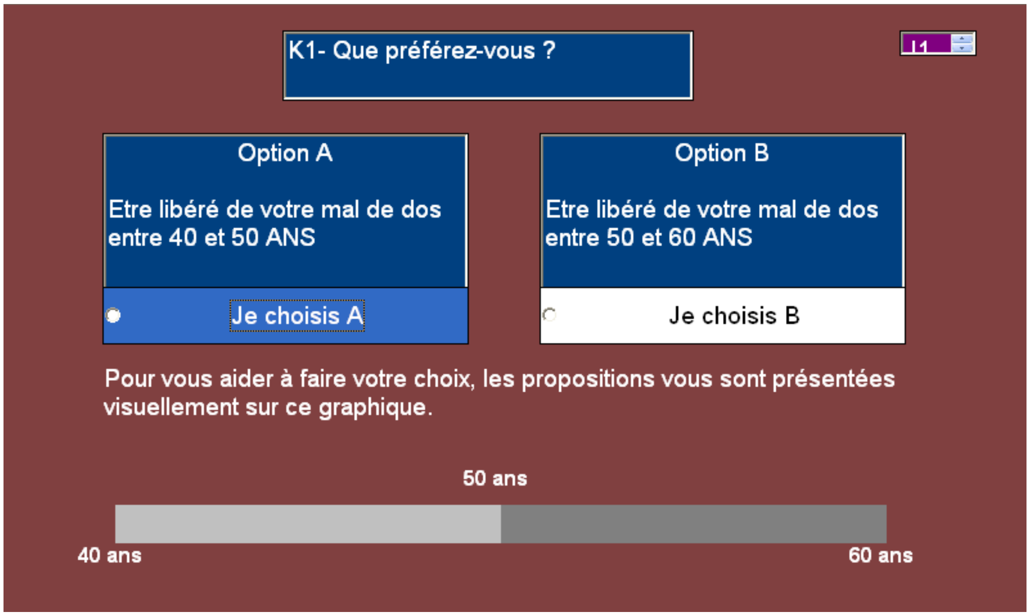

Figure A.2: Example of the original choice question for health, in French

B Rounding, iterations and indifferences

In this Section, we use the data from a selected participant to illustrate how we dealt with rounding. We then provide some descriptive statistics.

B.1 Data from a selected participant

Table B.1 shows the choices faced by subject ID1802401 and her/his answers in the elicitation of $t_{.5}$, for monetary outcomes. The value determined by three indifferences was equal to 42. In the analysis reported in the main text, the value was determined after four iterations and was equal to 43.

step	task	choice
step 1	$\alpha_{[38,48]}\beta$ vs. $\alpha_{[48,58]}\beta$	$\alpha_{[38,48]}\beta$
step 2	$\alpha_{[38,43]}\beta$ vs. $\alpha_{[43,58]}\beta$	$\alpha_{[38,43]}\beta$
step 3	$\alpha_{[38,41]}\beta$ vs. $\alpha_{[41,58]}\beta$	$\alpha_{[41,58]}\beta$
step 4	$\alpha_{[38,42]}\beta$ vs. $\alpha_{[42,58]}\beta$	$\alpha_{[42,58]}\beta$

Table B.1: Elicitation of $t_{.5}$ for subject ID1802401

Table B.2 shows the choices faced by subject ID1802401 and her/his answers in the elicitation of $t_{.75}$. The value determined by three indifferences was equal to 44. In the analysis conducted in the main text, the value was determined after four iterations and was equal to 45.

step	task	choice
step 1	$\alpha_{[43,51]}\beta$ vs. $\alpha_{[51,58]}\beta$	$\alpha_{[43,51]}\beta$
step 2	$\alpha_{[43,47]}\beta$ vs. $\alpha_{[47,58]}\beta$	$\alpha_{[43,47]}\beta$
step 3	$\alpha_{[43,45]}\beta$ vs. $\alpha_{[45,58]}\beta$	$\alpha_{[43,45]}\beta$
step 4	$\alpha_{[43,44]}\beta$ vs. $\alpha_{[44,58]}\beta$	$\alpha_{[44,58]}\beta$

Table B.2: Elicitation of $t_{.75}$ for subject ID1802401

Table B.3 shows the choices faced by subject ID1802401 and her/his answers for the elicitation of $t_{.875}$. The value determined by three indifferences was equal to 46 and the value determined by four indifferences was equal to 46.5.

step	task	choice
step 1	$\alpha_{[45,52]}\beta$ vs. $\alpha_{[52,58]}\beta$	$\alpha_{[45,52]}\beta$
step 2	$\alpha_{[45,49]}\beta$ vs. $\alpha_{[49,58]}\beta$	$\alpha_{[45,49]}\beta$
step 3	$\alpha_{[45,47]}\beta$ vs. $\alpha_{[47,58]}\beta$	$\alpha_{[45,47]}\beta$
step 4	$\alpha_{[45,46]}\beta$ vs. $\alpha_{[46,58]}\beta$	$\alpha_{[46,58]}\beta$

Table B.3: Elicitation of $t_{.875}$ for subject ID1802401

B.2 Descriptive statistics

Tables B.4 and B.5 show the elicited values for health and for money when only three indifferences were used.

	t.125	t.25	t.5	t.75	t.875
Median	2.00	4.00	9.00	14.00	16.50
Q1	0.00	0.00	0.00	3.00	4.75
Q3	4.50	8.00	12.00	17.00	18.50
Mean	4.75	6.06	8.40	11.99	13.36
Std	6.65	6.58	7.02	6.37	6.61

Table B.4: Summary of elicited values for health after three iterations

	t.125	t.25	t.5	t.75	t.875
Median	1.50	4.00	7.00	12.00	15.50
Q1	0.50	1.00	2.00	5.00	7.25
Q3	2.50	5.00	9.00	15.00	17.50
Mean	3.31	4.70	7.18	11.10	12.89
Std	5.21	5.24	5.90	5.68	6.15

Table B.5: Summary of elicited values for money after three iterations

Table B.6 shows the median and interquartile range for the areas for under the cumulative weighting functions, for both health and money. Kendall's tau was equal to 0.21.

	Health	Money
Median	0.54	0.58
Q1	0.41	0.51
Q3	0.88	0.80

Table B.6: Median areas under the cumulative weighting functions

Table B.7 shows the classification of subjects according to the value of the area under the cumulative weighting function.

	concave	linear	convex
concave	258	0	51
convex	122	1	63

Table B.7: Classification of subjects based on the shape of their cumulative weighting functions

Table B.8 shows the estimation results for constant, proportional, power, dual exponential and periodic discounting based on the median data.

	constant	proportional	power	dual exponential	periodic
Health	0.03	0.20	0.10	0.21	0.03
Money	0.06	0.10	0.41	0.11	0.36

Table B.8: Estimation results for the three discounting models, based on the median data. NLS routine was used for proportional discounting in the health domain due to non convergence of the residual sum of square minimization algorithm

C Continuous approximation of cumulative weights

C.1 Measurements from the direct method

The sequence of elicited values $(t_{.125}, t_{.25}, t_{.5}, t_{.75}, t_{.875})$ corresponds to a sequence of five (normalized) utilities C of life duration:

- $C(t_{.125}) = 1/8$,
- $C(t_{.25}) = 1/4$,
- $C(t_{.5}) = 1/2$,
- $C(t_{.75}) = 3/4$,
- $C(t_{.875}) = 7/8$.

To measure discounting, we used the relation between the utility of life duration C and the time weights for a constant outcome x. For a given value t_j, the direct method implies:

$$C(t_j) = \sum_{t=0}^{t_j} d_t.$$

By specifying a discount function d_t and using a continuous approximation, it is possible to elicit the underlying discounting parameter(s). Normalization implies $C(0) = 0$ and $C(T) = 1$.
A continuous version of the method implies:

$$C(t_j) = \int_{t=0}^{t_j} d_t dt.$$

The definition of the cumulative weight $C(t_j)$ implies :

$$C(t_j) - C(t_k).$$

is the average of the derivative C' over the interval $[t_k, t_j]$.

C.2 Constant discounting

The best-known case of d_t is constant discounting: time weights are equal to $e^{-\delta t}$, where δ is the discount rate. Therefore, using a continuous approximation:

$$C(t_j) = c \int_0^{t_j} e^{-\delta t} dt. \tag{1}$$

with c a normalization constant.
The primitive of $e^{-\delta t}$ is $-\frac{1}{\delta} e^{-\delta t}$. Then:

$$c \int_0^{t_j} e^{-\delta t} dt \quad = \quad c \left[-\frac{1}{\delta} e^{-\delta t} \right]_0^{t_j},$$

$$
\begin{aligned}
&= -\frac{c}{\delta}e^{-\delta t_j} + \frac{c}{\delta}, \\
&= \frac{c}{\delta}\left(1 - e^{-\delta t_j}\right).
\end{aligned}
$$

This gives $C(0) = 0$ and $C(T) = \frac{c}{\delta}\left(1 - e^{-\delta T}\right)$. Defining the normalization constant such that $C(T) = 1$:

$$c = \frac{\delta}{1 - e^{\delta T}},$$

one gets:

$$C[0, d] = \frac{1 - e^{-\delta t_j}}{1 - e^{-\delta T}}. \tag{2}$$

C.3 Proportional discounting

In Mazur's proportional discounting model, the time weights are equal to $\frac{1}{1+\kappa t}$, where κ is the parameter governing hyperbolic discounting. Therefore, using a continuous approximation:

$$C(t_j) = c\int_0^{t_j} \frac{1}{1+\kappa t}dt, \tag{3}$$

with c a normalization constant.
The primitive of $\frac{1}{1+\kappa t}$ is $\frac{ln(1+\kappa t)}{\kappa}$. Then:

$$
\begin{aligned}
c\int_0^{t_j} \frac{1}{1+\kappa t}dt &= \frac{c}{\kappa}\left[ln(1+\kappa t)\right]_0^{t_j}, \\
&= \frac{c}{\kappa}ln(1+\kappa t_j) - 0, \\
&= \frac{c}{\kappa}ln(1+\kappa t_j).
\end{aligned}
$$

This gives $C(0) = 0$ and $C(T) = \frac{c}{\kappa}ln(1 + \kappa T)$. Defining the normalization constant such that $C(T) = 1$:

$$c = \kappa\frac{1}{ln(1+\kappa T)},$$

one gets:

$$C(t_j) = \frac{ln(1+\kappa t_j)}{ln(1+\kappa T}. \tag{4}$$

C.4 Power discounting

Following Harvey's power discounting model, time weights are equal to $\frac{1}{(1+t)^\alpha}$. Therefore, using a continuous approximation:

$$C(t_j) = c\int_0^{t_j} (1+t)^{-\alpha}dt, \tag{5}$$

with c a normalization constant.
The primitive of $(1+t)^{-\alpha}$ is $\frac{(1+t)^{1-\alpha}}{1-\alpha}$. Then:

$$\begin{aligned} c\int_0^{t_j}(1+t)^{-\alpha}dt &= c\left[\frac{(1+t)^{1-\alpha}}{1-\alpha}\right]_0^{t_j}, \\ &= \frac{c}{1-\alpha}\left((1+t_j)^{1-\alpha}-1\right). \end{aligned}$$

This gives $C(0)=0$ and $C(T)=\frac{c}{1-\alpha}\left((1+T)^{1-\alpha}-1\right)$. Defining the normalization constant such that $C(T)=1$:

$$c=\frac{1-\alpha}{(1+T)^{1-\alpha}-1},$$

one gets:

$$C(t_j)=\frac{(1+t_j)^{1-\alpha}-1}{(1+T)^{1-\alpha}-1}. \tag{6}$$

C.5 Dual exponential discounting

Following Prelec and Rohde (2016), time weights are equal to $ae^{-r*t}+be^{r*t}+\zeta$. Therefore, using a continuous approximation:

$$C(t_j)=c\int_0^{t_j} ae^{-rt}+be^{rt}+\zeta dt. \tag{7}$$

With c a normalization constant.
The primitive of e^{-rt} is $-\frac{1}{r}e^{-rt}$ and the primitive of e^{rt} is $\frac{1}{r}e^{rt}$. Then:

$$W[0,d]=c.a.\underbrace{\int_0^{t_j} e^{-rt}dt}_{\left[-\frac{1}{r}e^{-rt}\right]_0^{t_j}}+c.b.\underbrace{\int_0^{t_j} e^{rt}+cdt}_{\left[\frac{1}{r}e^{rt}\right]_0^{t_j}}+c.\zeta\underbrace{\int_0^{d} dt}_{[t]_0^{t_j}}. \tag{8}$$

The equation can be rewritten:

$$C(t_j)=c\left(a.\left[-\frac{1}{r}e^{-rt}\right]_0^{t_j}+b.\left[\frac{1}{r}e^{rt}\right]_0^{t_j}+\zeta[t]_0^{t_j}\right). \tag{9}$$

$$C(t_j)=c\left(-\frac{a}{r}e^{-rt_j}+\frac{a}{r}+\frac{b}{r}e^{rt_j}-\frac{b}{r}+\zeta.t_j\right). \tag{10}$$

Assuming $a=-b=0.5$ and $\zeta=1$, one gets:

$$C(t_j)=\frac{c}{2r}.\left(t_j-e^{rt_j}-e^{-rt_j}\right). \tag{11}$$

This gives $C(0)=0$ and $C(T)=\frac{c}{2r}.\left(T-e^{rT}-e^{-rT}\right)$. Defining the normalization constant such that $C(T)=1$:

$$c=\frac{2r}{T-e^{rT}-e^{-rT}},$$

one gets:

$$C(t_j) = \frac{t_j - e^{rt_j} - e^{-rt_j}}{T - e^{rT} - e^{-rT}}. \qquad (12)$$

C.6 Periodic discounting

Following Prelec and Rohde (2016), time weights are equal to $\alpha cos(\rho t) + \beta sin(\rho t) + \gamma$. We normalize T to π. Therefore, using a continuous approximation:

$$\begin{aligned} C(t_j) &= t_j c. \int_0^{t_j<\pi} \alpha cos(\rho t) + \beta sin(\rho t) + \gamma dt, & (13)\\ &= c.\gamma \int_0^{t_j<\pi} dt + c.\alpha \int_0^{t_j<\pi} cos(\rho t) dt + c.\beta \int_0^{t_j<\pi} sin(\rho t) dt. & (14) \end{aligned}$$

With c a normalization constant.
The primitive of $sin(\rho t)$ is $-cos(\rho t)/\rho$ and the primitive of $cos(\rho t)$ is $sin(\rho t)/\rho$. Then:

$$C(t_j) = c\gamma[t]_0^{t_j} + \frac{c\alpha}{\rho}[sin(\rho t)]_0^{t_j} - \frac{c\beta}{\rho}[cos(\rho t)]_0^{t_j}. \qquad (15)$$

Then, for $t_j < \pi$:

$$C(t_j) = c\gamma t_j + \frac{c\alpha(sin(\rho t_j))}{\rho} - \frac{c\beta(cos(\rho t_j) - 1)}{\rho}. \qquad (16)$$

This gives $C(0) = 0$ and $C(T) = c\gamma T + \frac{c\alpha(sin(\rho T))}{\rho} - \frac{c\beta(cos(\rho T)-1)}{\rho}$. Defining the normalization constant such that $C(T) = 1$:

$$c = \frac{\rho}{\rho\gamma T + \alpha sin(\rho T) - \beta(cos(\rho T) - 1)},$$

one gets:

$$C(t_j) = \frac{\rho\gamma t_j + \alpha sin(\rho t_j) - \beta(cos(\rho t_j) - 1)}{\rho\gamma T + \alpha sin(\rho T) - \beta(cos(\rho T) - 1)}. \qquad (17)$$

with T normalized to π. We fix $\gamma = \alpha = -\beta = 0.5$, and get:

$$C(t_j) = \frac{\rho t_j + sin(\rho t_j) + (cos(\rho t_j) - 1)}{\rho T + sin(\rho T) + (cos(\rho T) - 1)}. \qquad (18)$$

D Empirical Cumulative Distribution Functions for Cumulative Weights

Figure D.1 shows the empirical cumulative distribution functions for cumulative weights $t_{.125}, t_{.25}, t_{.5}, t_{.75}$ and $t_{.875}$ for both health and money.

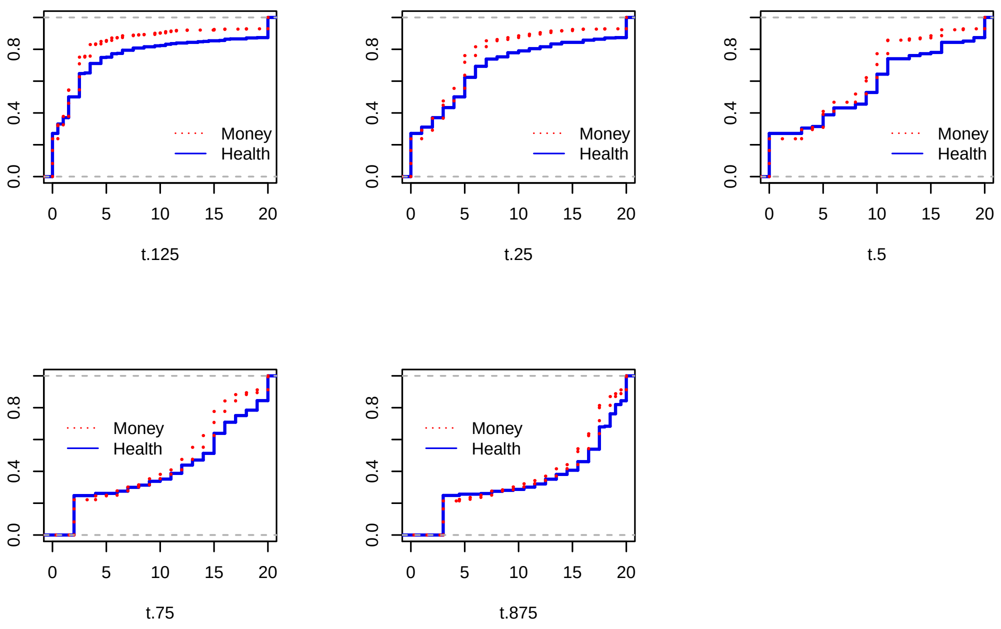

Figure D.1: Empirical Cumulative Distribution Functions for the Elicited Cumulative Weights

E Instructions and questionnaire

E.1 Experimental instructions (translated from French)

This survey aims to conduct research about the attitudes of individuals with regard to their quality of life, their health and their perception of subjective time. This study is conducted by the INPES (the French National Institute for Prevention and Education on Health) and INSERM (the French National Institute of Health and Medical Research). During this survey, your anonymity will be strictly protected, your contact information will be destroyed by the pool company (BVA /GFKISL) before communicating data to the INPES and INSERM, in accordance with the French law (Commission Nationale Informatique et Libertés). First, we will begin with a series of socio-demographic questions.

- Are you male or female?
- What is your age?
- What is your main occupation?
- Are you single or living as a couple?
- If you are living as a couple, what is your partner's main occupation?

We will now ask you a series of questions based on different scenarios. One scenario will be about health and quality of life. One scenario will be about your purchasing power. One scenario will be about your diet.

Imagine you suffer from back pain. Back pain corresponds to a mild but continuous pain. In practice this corresponds to:

- some problems walking about: unable to remain a long time in sitting or standing position, walking for a long time, unable to carry heavy loads.
- no problems with performing self care activities (e.g. eating, washing or dressing).
- some problems with performing usual activities (e.g. work, study, housework, family or leisure activities).
- mild pain or discomfort.
- not anxious or depressed.

Imagine that you suffer from back pain and that there is a treatment that allows complete relief. This treatment involves a weekly dose of pills, which would result in full health. Without the treatment, you will suffer again from the same above-mentioned back pain. Imagine that you have the possibility to take this treatment, but only for a given period of time.

We will use the computer to answer a series of questions. The health scenario is described on a cardboard card to which you can refer as often as you need during the series of questions.

Questions are about your preference between two periods of time when to take the treatment for complete back pain relief. Imagine that you can be relieved from back pain between your current age and your age+10 years, or between your current age+10 years and your current age +20 years. Of these two periods, in which one do you prefer to be released from back pain? Note that beyond this period you will still suffer from back pain. You will face a series of successive questions about your preference between two periods, with different lengths for the periods. Take your time to answer each of the questions. There are no right or wrong answers.

To help you express your preference, a strip placed at the bottom of the screen summarizes these two options. The light gray part corresponds to option A, whereas the dark gray portion corresponds to option B. If you choose option A (option B), it means that you will suffer from back pain during the time period in dark gray (light gray).

At the end of each series of questions, the first question will be repeated. If you answer differently to this repeated question, we will ask you to express your preferences again.

We will now ask you another series of questions relating this time to a scenario about purchasing power. Your purchasing power indicates what your income allows you to buy. Imagine your purchasing power is, for a given period of time, 20% higher compared to your purchasing power today and that without changing anything in your current activities (e.g without further work). This 20% increase in your purchasing power only occurs for a period of time. At the end of that period your purchasing power will return to its normal level.

Now we will use the computer to answer a series of questions. The purchasing power scenario is described on the following cardboard card to which you can refer as often as you need during the series of questions.

Questions are about your preference between two periods. Imagine your purchasing power can increase by 20% between your age and your age+10 years, or between your current age+10 years and your current age+20 years. Of these two periods, in which one do you prefer to have your purchasing power 20% higher compared to its current level? Note that beyond this period your purchasing power will be at the same level as it is now. You will face a series of successive questions about your preference between two periods, with different lengths for the periods. Take your time to answer to each of the questions. There are no right or wrong answers.

To help you express your preference, a strip placed at the bottom of the screen summarizes these two options. The light gray part corresponds to option A, whereas the dark gray portion corresponds to option B. If you choose option A (option B), it means that you have a 20% increase in your purchasing power during the time period in light gray (dark gray).

At the end of each series of question, the first questions will be repeated. If you answer differently to this repeated question, we will ask you to express your preferences again.

E.2 Additional questions and questionnaire

Subjects also faced a third scenario. This scenario was similar to the above-mentioned scenarios except that it was based on a choice between the two periods in which the subject was supposed to follow a low–salt diet allowing him to stay healthy in the future.

In addition to the scenarios, participants were asked a series of questions about their health status, their perception of the future, their tobacco and alcohol consumption, their perceptions of the risks associated with tobacco and alcohol consumption and their diet. The last series of questions was about their educational degree (if any), their financial situation and whether they sufferred from back pain or followed a low-salt diet.
